# Decisional impulsivity and the associative-limbic subthalamic nucleus in obsessive-compulsive disorder: stimulation and connectivity

**DOI:** 10.1093/brain/aww309

**Published:** 2016-12-30

**Authors:** Valerie Voon, Fabien Droux, Laurel Morris, Stephan Chabardes, Thierry Bougerol, Olivier David, Paul Krack, Mircea Polosan

**Affiliations:** 1 Department of Psychiatry, University of Cambridge, Addenbrooke’s Hospital, Cambridge CB2 0QQ, UK; 2 Cambridgeshire and Peterborough NHS Foundation Trust, Cambridge, UK; 3 Univ. Grenoble Alpes, Inserm U1216 Grenoble Institute of Neuroscience, CHU Grenoble Alpes, F-38000 Grenoble, France; 4 Department of Clinical Neurosciences; Faculty of Medicine, University of Geneva, Geneva, Switzerland

**Keywords:** obsessive-compulsive disorder, deep brain stimulation, subthalamic nucleus, impulsivity, uncertainty

## Abstract

Why do we make hasty decisions for short-term gain? Rapid decision-making with limited accumulation of evidence and delay discounting are forms of decisional impulsivity. The subthalamic nucleus is implicated in inhibitory function but its role in decisional impulsivity is less well-understood. Here we assess decisional impulsivity in subjects with obsessive compulsive disorder who have undergone deep brain stimulation of the limbic and associative subthalamic nucleus. We show that stimulation of the subthalamic nucleus is causally implicated in increasing decisional impulsivity with less accumulation of evidence during probabilistic uncertainty and in enhancing delay discounting. Subthalamic stimulation shifts evidence accumulation in subjects with obsessive-compulsive disorder towards a functional less cautious style closer to that of healthy controls emphasizing its adaptive nature. Thus, subjects with obsessive compulsive disorder on subthalamic stimulation may be less likely to check for evidence (e.g. checking that the stove is on) with no difference in subjective confidence (or doubt). In a separate study, we replicate in humans (154 healthy controls) using resting state functional connectivity, tracing studies conducted in non-human primates dissociating limbic, associative and motor frontal hyper-direct connectivity with anterior and posterior subregions of the subthalamic nucleus. We show lateralization of functional connectivity of bilateral ventral striatum to right anterior ventromedial subthalamic nucleus consistent with previous observations of lateralization of emotionally evoked activity to right ventral subthalamic nucleus. We use a multi-echo sequence with independent components analysis, which has been shown to have enhanced signal-to-noise ratio, thus optimizing visualization of small subcortical structures. These findings in healthy controls converge with the effective contacts in obsessive compulsive disorder patients localized within the anterior and ventral subthalamic nucleus. We further show that evidence accumulation is associated with anterior associative-limbic subthalamic nucleus and right dorsolateral prefrontal functional connectivity in healthy controls, a region implicated in decision-making under uncertainty. Together, our findings highlight specificity of the anterior associative-limbic subthalamic nucleus in decisional impulsivity. Given increasing interest in the potential for subthalamic stimulation in psychiatric disorders and the neuropsychiatric symptoms of Parkinson’s disease, these findings have clinical implications for behavioural symptoms and cognitive effects as a function of localization of subthalamic stimulation.

## Teaser

The subthalamic nucleus (STN) has been implicated in impulse control. Voon *et al*. report that the associative-limbic STN is involved in hasty decisions for short-term gain. STN stimulation in obsessive-compulsive disorder shifts individuals towards less cautious decision making. Limbic and motor function and connectivity are dissociable in the human STN.

## Introduction

Why do we make hasty decisions, choosing short-term gains? Self-control, or impulsivity, is heterogeneous with decisional and motor subtypes, with overlapping yet distinct neural networks (Voon and Dalley, 2016). Both hasty decisions (accumulating limited evidence prior to a decision) and choosing short term smaller rewards over longer term larger rewards are forms of decisional impulsivity also known as reflection impulsivity and delay discounting, respectively.

The subthalamic nucleus (STN) plays a crucial role in inhibitory function mediating the switch from automatic to controlled responding (Hikosaka and Isoda, 2010). The STN is uniquely placed as a relay structure within the indirect pathway and receives hyperdirect signals from cortical regions (Nambu *et al.*, 2002). The STN, similar to parcellation of motor, cognitive and limbic fronto-striatal circuitry, also shows functional specificity (Haynes and Haber, 2013). Its causal role can be assessed in patients who have undergone STN deep brain stimulation (DBS), which involves high frequency stimulation via electrodes inserted into grey or white matter targets to modulate network activity or pathological oscillatory activity. STN DBS targeting the motor STN is effective for the management of Parkinson’s disease. STN DBS targeting the limbic and associative STN has also been shown to be effective in a randomized controlled trial for obsessive compulsive disorder (OCD) (Mallet *et al.*, 2008).

The STN has been implicated in impulse control and is most consistently associated with early responding and lower evidence accumulation in the context of conflict or competing responses (Jahanshahi *et al.*, 2000; Schroeder *et al.*, 2002; Frank *et al.*, 2007; Green *et al.*, 2013). STN DBS also has a mixed effect on response inhibition with greater impairments as a function of prepotency of response bias (Hershey *et al.*, 2004), task difficulty (Green *et al.*, 2013), baseline status (Ray *et al.*, 2009) and early responses dissociable from a late inhibitory process (Green *et al.*, 2013). The effects of STN DBS on decisional impulsivity are less well-understood. Lesions of the STN in rodents decrease delay discounting (Winstanley *et al.*, 2005; Uslaner and Robinson, 2006) (improving impulsivity) but STN DBS in patients with Parkinson’s disease targeting the motor STN has not been shown to influence delay discounting (Evens *et al.*, 2015; Seinstra *et al.*, 2016). Here we focus on evidence accumulation during probabilistic inference, which can be tested using the Beads in a Jar task (Beads Task). Subjects view two jars with opposing proportions of coloured beads and must decide from which jar sequentially displayed beads are selected. The number of beads selected prior to decision provides an index of reflection impulsivity. Reflection impulsivity using the Beads Task is associated with dorsolateral prefrontal, parietal and anterior insular function and volumetric differences (Stern *et al.*, 2010; Banca *et al.*, 2016). Patients with Parkinson’s disease who have undergone DBS targeting the motor STN do not show any differences in reflection impulsivity tested using the Beads Task compared to Parkinson’s disease controls (Djamshidian *et al.*, 2013*a*). The role of DBS targeting the associative-limbic STN in these forms of decisional impulsivity is not yet known.

OCD is characterized by obsessions or repetitive intrusive thoughts and urges leading to compulsions or behaviours that subjects feel driven to perform. OCD is commonly associated with impairments in motor impulsivity, specifically impaired response inhibition or action cancellation after motor initiation, as measured using the Stop Signal task (Menzies *et al.*, 2007). Other forms of impulsivity are less consistently observed. Delay discounting was shown to be enhanced in OCD in one large study (*n* = 80 patients) (Sohn *et al.*, 2014) but these findings have not been shown across all studies (Pinto *et al.*, 2014). OCD patients have consistently shown enhanced error-related negativity relative to healthy controls (Gehring *et al.*, 2000; Johannes *et al.*, 2001) in response to conflict tasks with more variability in behavioural differences (Ursu *et al.*, 2003; Fitzgerald *et al.*, 2005; Page *et al.*, 2009; Najmi *et al.*, 2010; Kashyap *et al.*, 2013; Marsh *et al.*, 2014). This error-processing enhancement is localized within the rostral anterior cingulate (Kiehl *et al.*, 2000; Fitzgerald *et al.*, 2005) with similar enhanced activity also during correct high conflict trials, suggesting abnormalities in conflict resolution in OCD (Ursu *et al.*, 2003; Endrass *et al.*, 2008). We have previously shown using the random dot motion task, enhanced evidence accumulation (longer reaction times or lower impulsivity) to greater uncertainty or conflict in OCD on both behavioural and computational modelling (Banca *et al.*, 2015). In this task, subjects must decide whether randomly moving dots are more likely to be moving left or right, thus representing evidence accumulation under perceptual uncertainty. Using the Beads Task, evidence accumulated during probabilistic uncertainty has been shown to be higher in OCD (Fear and Healy, 1997; Pelissier and O’Connor, 2002), although these findings are not always consistent (Grassi *et al.*, 2015) or only significant after controlling for neuroticism (Volans, 1976). This tendency to accumulate evidence or check for evidence has been linked to enhanced doubt or subjective confidence or uncertainty. Uncertainty (e.g. the possibility of alternative outcomes) has been suggested to induce OCD subjects to gather excessive evidence (e.g. obsessional rumination and checking) to support their decision. Some (Dar, 2004; Stern *et al.*, 2013) but not all studies (Sarig *et al.*, 2012; Banca *et al.*, 2014) have shown impairments in subjective certainty in OCD. Using a delayed matching-to-sample task with unrestricted choice verification, poor insight triggered greater checking behaviours in OCD patients, which indexed uncertainty (Rotge *et al.*, 2008; Jaafari *et al.*, 2011). OCD subjects have also shown greater explicit subjective ratings of uncertainty for low but not higher uncertainty evidence in a probabilistic reasoning task (Stern *et al.*, 2013).

In the first study, we assess the causal role of the STN by asking whether STN DBS targeting the associative-limbic STN in OCD patients is associated with two different forms of decisional impulsivity: reflection impulsivity or evidence accumulation using the Beads Task and delay discounting using the Monetary Choice Questionnaire. In the second separate study in healthy control subjects, we map resting state functional connectivity of limbic, cognitive and motor prefrontal regions to the STN based on known hyper-direct tract tracing studies in non-human primates. We also ask whether resting state functional connectivity of anterior associative-limbic or posterior motor STN seeds to prefrontal regions is associated with decisional impulsivity. We use a recently developed multi-echo sequence with independent components analysis (ME-ICA), which enhances signal-to-noise ratio for enhanced visualization of small subcortical structures (Kundu *et al.*, 2013) and greater sensitivity to functional network measures relative to single echo (Baek *et al.*, 2016). We hypothesize that DBS of the anterior associative-limbic STN enhances reflection impulsivity but decreases delay discounting. We further hypothesize that resting state functional connectivity of the antero-medial STN is associated with decisional impulsivity.

## Materials and methods

### Participants

#### Study 1

Twelve OCD subjects were recruited from Grenoble University Hospital, tested on and off DBS and compared with healthy volunteers. OCD patients (eight females; 41.75 ± 7.94 years old) had undergone bilateral STN DBS 38.1 ± 18.8 months prior to testing. Patient characteristics are described in Table 1. Before surgery, average disease duration was 18 ± 9.2 years, and Yale Brown Obsessive Compulsive Scale (YBOCS) score was 34.3 ± 3.2. At the time of the study, YBOCS baseline score was 20 ± 9.1 with a clinical improvement of 41 ± 28%. Patients had at least 5 years of treatment-resistant, severe, disabling OCD before DBS. All patients underwent STN DBS for at least 5 months prior to the study (range: 5–71 months). One subject had comorbid Tourette’s syndrome and another subject had comorbid skin picking. One subject had a premorbid history of an eating disorder that was in remission 20 years before surgery.

**Table 1:** Clinical and demographical characteristics of the OCD patients

Patient number/age (years)/gender (F/M)	Age at surgery (years)	Duration of disease before surgery (years)	Age at onset of OCD (years)	Duration of DBS (months)	YBOCS before surgery	YBOCS baseline at time of study	Medications at the time of the study
1/46/M	39	18	21	71	37	25	Fluvoxamine 200 mg/day
							Lorazepam 4 mg/day
2/49/F	42	25	17	64	30	28	Aripiprazole 30 mg/day
							Olanzapine 5 mg/day
							Escitalopram 20 mg/day
							Clomipramine 75 mg/day
3/39/M	36	17	19	32	32	28	Paroxetine 60 mg/day
4/53/F	49	39	10	51	35	29	Fluoxetine 20 mg/day
							Clomipramine 25 mg/day
5/37/M	34	13	21	22	32	27	Clomipramine 150 mg/day
							Oxazepam 175 mg/day
							Alimemazine 50 mg/day
6/41/F	38	11	27	35	36	6	None
7/43/F	40	15	25	32	36	23	Fluvoxamine 200 mg/day
							Hydroxyzine 50 mg/day
							Clomipramine 25 mg/day
8/41/F	37	5	32	44	32	2	Venlafaxine 37.5 mg/day
							Clotiazepam 10 mg/day
9/30/M	27	10	17	25	38	24	Sertraline 50 mg/day
							Aripiprazole 20 mg/day
							Methylphenidate 60 mg/day
							Pitolisant 20 mg/day
10/56/F	52	25	27	51	40	18	Zopiclone 7.5 mg/day
							Aripiprazole 2.5 mg/day
							Hydroxyzine 100 mg/day
11/33/F	33	21	12	5	30	11	Venlafaxine 150 mg/day
12/33/F	33	26	7	25	34	19	Fluoxetine 20 mg/day
							Levothyroxine 125 µg/day

M = male; F = female.

The OCD subjects were implanted bilaterally with two electrodes 3389 connected to a Kinetra stimulator (Medtronic), accordingly to the STN DBS protocol already published elsewhere (Mallet *et al.*, 2008; Chabardes *et al.*, 2013). The procedure targeted the antero-medial non-motor part of the STN. Indirect targeting was defined as 1 mm anterior to the mid-commissural point, 10 mm lateral from the midline and 4 mm below the AC-PC line. The final target was adapted laterally according to the visualization of the medial border of the STN. The antero-posterior coordinates were defined 2 mm anterior to the anterior border of the red nucleus. Stimulation frequency and pulse width were set at 130 Hz and 60 µs, respectively; stimulation voltage and activated contacts were adjusted individually to obtain the best clinical response.

The 24 age- and gender-matched healthy control subjects (16 females; 42.67 ± 8.34 years old) were recruited from the University and community in Grenoble. Subjects were interviewed by a psychiatrist, and screened with the Structured Clinical Interview for the Diagnostic and Statistical Manual of Mental Disorders, Version IV, to check the exclusion criteria. Exclusion criteria for healthy volunteers were past or present serious psychiatric or medical disorders as well as any psychotropic medications. The research protocol was approved by the Ethics Committee of Grenoble University Hospital (ancillary study to protocol N° ID RCB: 2012-A00490-43). All participants volunteered to participate in the study and gave written informed consent.

#### Study 2

Healthy volunteers (*n* = 154) were recruited from the University of Cambridge and community-based advertisements in East Anglia. Subjects aged 18 and over were tested and were excluded if they had a major psychiatric disorder, substance addiction, a history of regular or current use of substances, serious medical illness or were on psychotropic medications, and screened with the Mini International Neuropsychiatric Interview (Sheehan *et al.*, 1998).

Subjects provided written informed consent before participating. Participants were compensated for their time. The study was approved by the University of Cambridge Research Ethics Committee.

### Study design

#### Tasks

**Figure 1:**
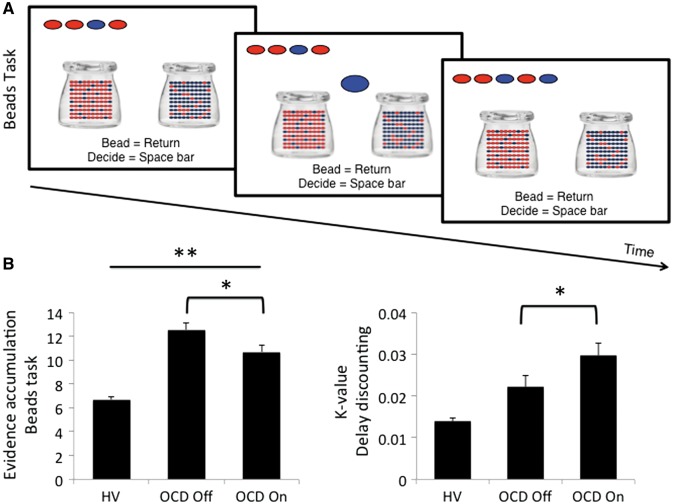
**Beads Task and primary outcomes.** (**A**) Beads Task: subjects viewed two jars with opposing ratios of red and blue beads (*P* = 0.80; *P* = 0.20). Based on the sequential viewing of beads selected from a jar, participants were asked to make a decision from which jar the beads were selected. The selected beads were shown at the top to control for working memory effects. (**B**) Evidence accumulated (number of beads prior to decision) in healthy volunteers (HV), OCD subjects on and off DBS targeting the subthalamic nucleus. Error bars represent standard error of the mean. *Related-samples Wilcoxon Signed Rank Test, *P* < 0.05; **Independent samples Kruskal Wallis test, *P* < 0.01 with *post hoc* differences between healthy volunteers and OCD subjects off DBS (*P* < 0.005).

In both studies subjects completed the same tasks. Reflection impulsivity was assessed with the Beads Task. Subjects were shown two jars on the computer screen with opposite ratios of red and blue beads (*P* = 0.80; *P* = 0.20) (Fig. 1). They were informed of the bead ratio and were told that beads from one of the jars would be presented one at a time in the centre of the screen. The drawn beads were shown at the top of screen to control for working memory effects. The subjects’ goal was to infer from which jar the beads were drawn. Subjects were free to view as many beads as they wanted without time limit up to 20 before committing to their decision. Subjects pressed the ‘Return’ key to view more beads and the ‘Space bar’ when they were ready to make a decision. Following their decision, they then indicated the degree of confidence that their answer was correct on a visual analogue scale anchored at ‘Not confident’ to ‘Very confident’ using a mouse. There was no feedback. Subjects were then informed that the next block would start. The primary outcome measure was the number of beads drawn prior to a decision. Other outcome measures included confidence ratings and objective probability at the time of decision. There were three blocks of trials with the same bead order (Banca *et al.*, 2015).

Subjects also completed the Monetary Choice Questionnaire choosing between small immediate and larger delayed rewards. The primary outcome measure was the discount parameter K.

#### Study 1

Patients performed the tasks with STN DBS on and off, in a randomized double-blind within-subject design over two successive days to allow a sufficiently long washout of DBS effects. Patients were randomized to either one of two arms: on Day 1, DBS was kept on (switched off) and patients tested 4 h later; DBS was kept on (off) overnight. On Day 2, DBS was switched off (on) early in the morning and patients tested 4 h later. If necessary, patients stayed in hospital both nights. The DBS control device was manipulated by another investigator to keep the tester and patient blind. Patients continued their usual medication during the protocol. Subjects also completed the Spielberger State Anxiety Inventory both on and off DBS.

#### Statistical analysis

Data were inspected for outliers [>3 standard deviation (SD) from group mean] and normality of distribution using Shapiro-Wilkes test. Although the number of beads accumulated was normally distributed, the delay discounting measure was not normally distributed; non-parametric tests were used for all parameters. Related samples Wilcoxon Signed Rank Test was used to compare on and off DBS; independent samples Kruskal Wallis test was used to compare more than two groups and independent samples Mann-Whitney U-test for *post hoc* analyses if the Kruskal Wallis test was significant. The independent samples Mann-Whitney U-test was also used to compare order effects. Clinical variables of YBOCS on DBS prior to the experiment and preoperative YBOCS were correlated with primary and secondary outcome measures (Bonferroni correction *P* ≤ 0.003 for 16 observations). The relationship between evidence accumulation and probability or confidence was examined using linear and quadratic curve estimation model fits. Probability was calculated using Bayesian analysis to estimate on a trial-by-trial basis based on the accumulated evidence the likelihood that the correct jar was actually the correct jar.

#### Study 2

The resting state sequence and analysis was conducted with ME-ICA, which has enhanced signal-to-noise ratio thus enabling visualization of small subcortical structures (Kundu *et al.*, 2013). Resting state functional MRI data were collected during rest for 10 min with eyes open with a Siemens 3 T Tim Trio scanner with 32-channel head coil at the Wolfson Brain Imaging Centre, University of Cambridge (repetition time, 2.47 s; flip angle, 78°; matrix size 64 × 64; in-plane resolution, 3.75 mm; field of view, 240 mm; 32 oblique slices, alternating slice acquisition slice thickness 3.75 mm with 10% gap; iPAT factor, 3; bandwidth = 1.698 Hz/pixel; echo time = 12, 28, 44 and 60 ms). Anatomical T_1_-weighted magnetization prepared rapid gradient echo (MPRAGE) (176 × 240 field of view; 1-mm in-plane resolution; inversion time, 1100 ms) data were also acquired.

Multi-echo independent component analysis (ME-ICA v2.5 beta10; http://afni.nimh.nih.gov) was used to de-noise functional data. Data were decomposed into independent components using FastICA and independent components that strongly scaled with echo time were retained as blood oxygen level-dependent (BOLD) data (Kundu *et al.*, 2013). Echo time-independent components were assigned as non-BOLD artefacts and were removed by projection, robustly de-noising data for motion, physiological and scanner artefacts based on physical principles. De-noised echo planar images were co-registered with their anatomical MPRAGE data and normalized to a standard Montreal Neurological Institute (MNI) template. Spatial smoothing was performed with a Gaussian kernel full-width half-maximum = 6 mm, except for baseline mapping of STN connectivity, which was left unsmoothed.

First, baseline functional connectivity between ventral striatum and posterior putamen, as well as cortical regions, with STN was examined to dissociate functionally distinct STN regions. We used functionally-defined prefrontal cortical regions for regions of interest which we had previously used to define fronto-striatal circuitry (Morris *et al.*, 2016b) and which paralleled primate anterograde tract tracing studies demonstrating known hyperdirect projections to STN (Haynes and Haber, 2013) {e.g. dorsolateral prefrontal cortex [dlPFC; Brodmann area (BA) 9 and 46]; dorsal cingulate (dACC; BA 24); pre-supplementary motor area (pre-SMA; Rrostral BA 6); SMA (caudal BA 6) and M1 (BA4)}. Functional connectivity was computed using a seed-driven approach using the CONN-fMRI Functional Connectivity toolbox (Whitfield-Gabrieli and Nieto-Castanon, 2012) for SPM. Functional data were temporally band-pass filtered (0.008 < f < 0.09 Hz) and significant principle components of white matter and CSF were removed. Significance was assessed with small volume corrected (SVC) for STN, family wise error (FWE) *P* < 0.05.

Secondly, to assess the relationship between anterior and posterior STN with the behavioural measures, anterior STN was dissociated based on ventral striatal and dlPFC connectivity with whole STN. Both ventral striatum and dlPFC showed connectivity with anterior STN (ventral striatum to right STN; dlPFC to bilateral STN) had a posterior extent at *y* = −14. As both regions of interest had considerable overlap, we thus combined limbic and associative anterior STN and divided this from motor posterior STN by dissecting the anterior STN at *y* = −14, dividing STN into anterior and posterior subregions (Figs 3 and 4). Both anterior and posterior STN seed-to-whole brain functional connectivity maps were computed with primary outcome measures used as covariates. As the K-value was not normally distributed, the K-values were log10 transformed. Of the 154 healthy volunteers, 45 completed the Beads Task and 76 completed the Monetary Choice Questionnaire. Whole brain cluster-extent threshold FWE *P* < 0.05 was considered significant.

All procedures contributing to this work comply with the ethical standards of the relevant national and institutional committees on human experimentation and with the Declaration of Helsinki.

## Results

### Study 1

One OCD subject off DBS did not complete the Beads Task and was not included. In the primary outcome measures, STN DBS on versus off in OCD subjects was associated with less evidence accumulated (fewer beads, greater impulsivity) (related-samples Wilcoxon Signed Rank Test, *P* = 0.04) and greater impulsive choice (greater delay discounting) (*P* = 0.03) (Fig. 1B). In secondary outcome measures, there were no differences on versus off DBS in subjective confidence (*P* = 0.53) or probability at the time of decision (*P* = 0.79). There were no differences in the anxiety measure on versus off DBS (on: 45.40 ± 10.27; off: 45.30 ± 12.07; *P* > 0.1).

In the comparison with healthy controls, there was an overall group difference in evidence accumulated on the Beads Task [healthy volunteers 5.49 (SD 2.66); OCD off 12.55 (SD 6.72); OCD on 10.69 (SD 7.16); independent-samples Kruskal Wallis test, *P* = 0.007] (Fig. 1B). *Post hoc* analyses showed that OCD subjects off DBS (independent samples Mann-Whitney U-test; *P* = 0.002) but not on DBS (*P* = 0.08) accumulated more evidence than healthy controls. There were no differences in delay discounting [healthy volunteers 0.014 (SD 0.016); OCD off 0.022 (SD 0.027); OCD on 0.030 (SD 0.030); *P* = 0.232] (Fig. 1B) or in subjective confidence [healthy volunteers 363.15 (SD 80.47); OCD off 355.8 (SD 98.11); OCD on 336.22 (SD 97.33); *P* = 0.531] or probability [healthy volunteers 0.89 (SD 0.12); OCD off 0.85 (SD 0.16); OCD on 0.88 (SD 0.13); *P* = 0.898].

**Figure 2:**
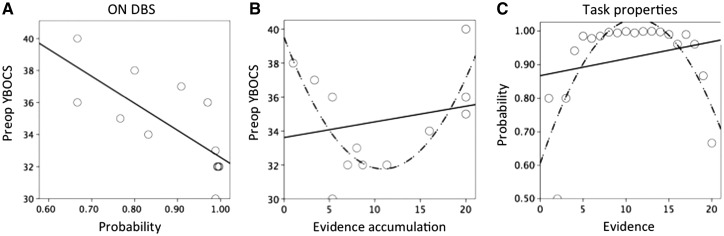
**Evidence accumulation and presurgical severity.** (**A**) Preoperative OCD severity (YBOCS) in OCD subjects on DBS is negatively linearly associated with objective probability at the time of decision. (**B**) This relationship with objective probability was related to differing responding styles (low or high evidence accumulation, both associated with lower probability). (**C**) For illustration purposes, the objective probability of the task (i.e. that the correct jar was correct based on the available evidence) is plotted for each trial averaged across the three blocks. Model fits and *P*-values are reported in the text.

Severity of YBOCS prior to surgery, or an index of preoperative OCD severity, was negatively linearly correlated with probability at the time of decision on DBS (Spearman Rank = −0.79, *P* = 0.002) (Fig. 2A) but no other correlations were observed. YBOCS prior to the experiment (on DBS) was not linearly correlated with outcomes (*P* > 0.05).

To understand the relationship observed between preoperative YBOCS and probability, the relationship between preoperative YBOCS and evidence accumulation was plotted revealing a quadratic relationship (linear: R^2^ = 0.05; *P* = 0.50; quadratic: R^2^ = 0.61, *P* = 0.014) (Fig. 2B). Subjects with high preoperative OCD severity either had low or high evidence accumulation thus accounting for the negative linear relationship with probability.

For illustration purposes, the relationship between actual evidence and objective probability in the task was also examined, which was shown to be quadratic (linear: R^2^ = 0.05, *P* = 0.345; quadratic: R^2^ = 0.65, *P* < 0.0001) (Fig. 2C). Probability was estimated on a trial-by-trial basis based on the actual evidence to illustrate the likelihood that the correct jar was actually the correct jar. Thus, objective probability can shift across trials depending on the evidence. In the task design, two blocks became increasingly more uncertain with decreasing probability with increasing number of trials (e.g. in one block, initially the majority of trials were red beads in the first 10 blocks with increasing blue beads in the next 10 blocks) hence resulting in a quadratic relationship in which objective probability in the task decreased at the extremes with either fewer or more trials.

There were no order effects (independent samples Mann-Whitney U-test, *P* > 0.05) or relationship between the primary outcome measures.

### Study 2

#### Baseline STN connectivity

**Figure 3:**
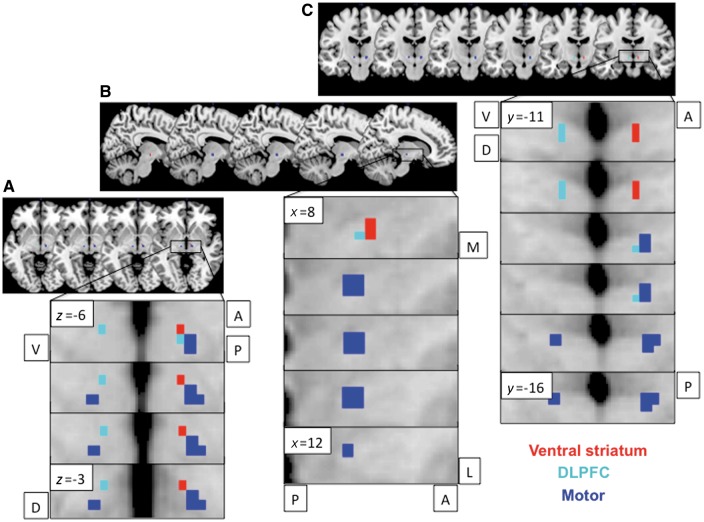
**Limbic-associative and motor connectivity dissociating anterior and posterior STN.** Resting state functional connectivity of ventral striatal (red) and dorsolateral prefrontal cortex (DLPFC; cyan) seeds to anterior STN and primary motor cortex (blue) seed to posterior STN shown for axial (**A**), sagittal right (**B**) and coronal (**C**) STN slices. Bilateral ventral striatal seeds showed lateralized functional connectivity to right STN. The ventral striatal and dlPFC activations are shown at FWE *P* < 0.05 and motor activations are shown at FWE *P* < 0.001 with an STN mask on a standard MNI template. The different thresholds were used for illustration purposes as motor activity at lower threshold otherwise activated the entire STN. A = anterior; P = posterior; V = ventral; D = dorsal.

**Figure 4:**
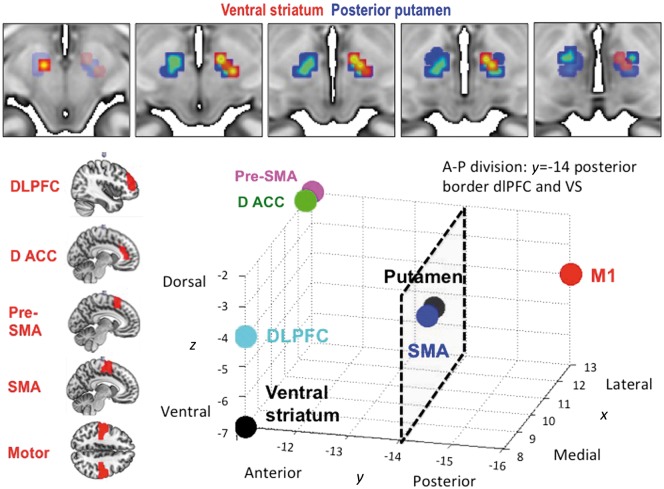
**Activity and peak coordinates in the STN of resting state functional connectivity from limbic-associative and motor regions of interest in healthy controls.***Top*: Ventral striatal (red) and posterior putamen (blue) seed regions of interest were used to compute resting state functional connectivity within STN (small volume corrected for STN FWE *P* < 0.05). The ventral striatal activations are shown at FWE *P* < 0.05 and putaminal activation at FWE *P* < 0.001 with an STN mask on a standard MNI template. The different thresholds were used for illustration purposes as putaminal activity at lower threshold otherwise activated the entire STN. *Bottom*: The left figures show the seed regions of interest. The 3D plot shows the peak voxels for seed regions of interest correlating with STN. Ventral striatum (VS) had a peak in right anterior STN (*y* = −11); dlPFC and dACC had peaks bilaterally in anterior STN (*y* = −11); and SMA and primary motor cortex had peaks bilaterally in posterior STN (*y* = −14 to −16). The anterior-posterior (A-P) division used to divide anterior associative-limbic and posterior motor STN to examine behavioural correlates were based on the posterior border of dlPFC and ventral striatum at *y* = −14. Note that the plots for pre-SMA and dACC and for putamen and SMA overlap but for illustration purposes are shown separated by *x* = 0.5 mm.

**Figure 5:**
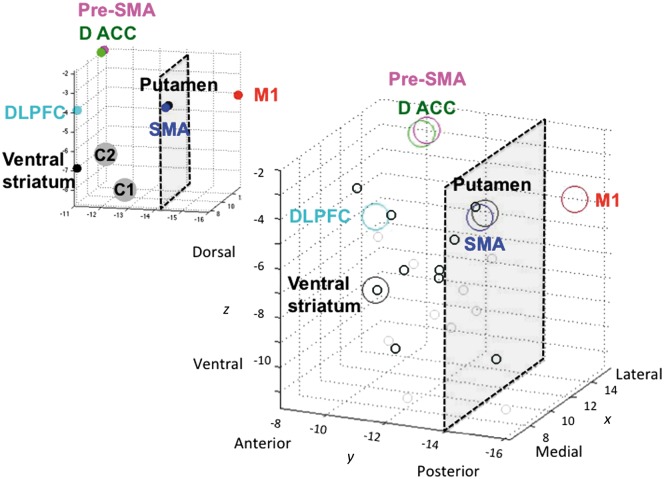
**Peak coordinates in the STN of resting state functional connectivity from limbic-associative and motor regions of interest in healthy controls.** The relationship to optimal DBS contacts from patients with obsessive compulsive disorder in Study 1 are also shown. Resting state functional connectivity for striatal and cortical seed regions of interest and peak connectivity within STN for healthy controls are plotted as per Fig. 4. The *top left* graph shows these peaks in relation to the averaged coordinates for Contact 1 (C1) and Contact 2 (C2) (actual coordinates in *x y z* in mm averaged for C1 and C2 across the OCD patients from Study 1; the second and third from the four contacts C0 to C3), contacts from which optimal stimulation targeting obsessive compulsive symptoms was achieved. The *bottom right* graph shows C1 (small light grey circle) and C2 (small dark grey circle) plotted for each patient.

**Figure 6:**
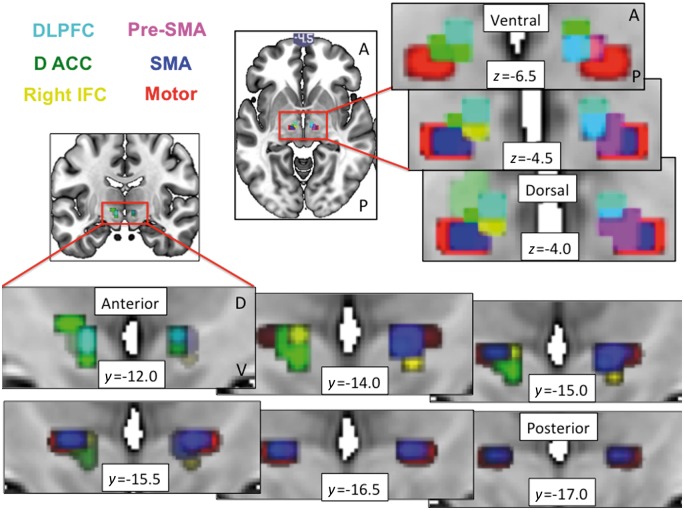
**Overlay of limbic-associative and motor cortical connectivity to STN.** Limbic-associative and motor cortical seeds to STN resting state functional connectivity are overlaid and shown as serial coronal (*left* and *bottom*) and axial STN images (*top* and *right*). The dlPFC and inferior frontal cortex (IFC) were shown at FWE *P* < 0.05 and dACC, pre-SMA and SMA shown at FWE *P* < 0.001 using an STN mask on a standard MNI template. The different thresholds were used for illustration purposes as dACC, pre-SMA and SMA activity at lower threshold otherwise activated the entire STN.

Resting state functional MRI data were collected from 154 healthy volunteers (71 female; age = 31.3 ± 12.842 years). We replicate previous findings (Morris *et al.*, 2016a) of limbic and associative anterior and motor posterior subregions of STN, based on ventral striatum and posterior putamen functional connectivity, respectively (Figs 3 and 4). The peak values for the ventral striatum, dlPFC and dorsal ACC connectivity to STN were more anterior bilaterally (*y* = −11) whereas SMA and M1 were more posterior bilaterally (*y* = −14 to −16) (the peak connectivity within the right STN are plotted on a 3D axis for visualization in Fig. 4). In particular, we show that ventral striatum had right-sided unilateral connectivity with STN, with a unilateral peak in ventromedial anterior right STN (right STN: Z = 2.92, *x y z* = 8 − 11 − 7) with left STN not significant at SVC FWE *P* < 0.05 (Fig. 3). Posterior putamen had more widespread connectivity with STN with the peak in right STN in posterior STN but in left STN in anterior STN (right STN: Z = 4.25, *x y z* = 10 − 14 − 4; left STN: Z = 3.99, *x y z* = − 10 − 11 − 7) (Figs 3–6). DLPFC was dissociable on an anterior-posterior axis, with peaks bilaterally in anterior STN (right STN: Z = 2.40, *x y z* = 8 − 11 − 4; left STN: Z = 2.16, *x y z* = − 8 − 11 − 7) and the posterior extent at *y* = −14. Dorsal ACC and SMA had the strongest and most widespread functional connectivity with STN with peaks in anterior and posterior bilateral STN respectively (dACC: right STN: Z = 4.92, *x y z* = 13 − 11 − 2; left STN: Z = 5.39, *x y z* = − 13 − 11 − 2; SMA: right STN: Z = 5.32, *x y z* = 10 − 14 − 4; left STN: Z = 4.28, *x y z* = − 13 − 16 − 4), followed by primary motor cortex (M1) with peaks in posterior bilateral STN (right STN: Z = 4.33, *x y z* = 13 − 16 − 4; left STN: Z = 4.50, *x y z* = − 10 − 16 − 4) (Figs 3–6). The pre-SMA and right IFC, both nodes within the ‘stopping’ network, had peaks overlapping across both anterior and posterior STN (pre-SMA: right STN: Z = 3.94, *x y z* = 13 − 11 − 2; left STN: Z = 2.17, *x y z* = − 10 − 16 − 9; right IFC: right STN: Z = 2.89, *x y z* = 10 − 16 − 9). Dorsomedial PFC and orbitofrontal cortex showed no significant findings.

The cortical and striatal functional connectivity peaks to STN in healthy controls are contrasted with actual effective DBS contacts (coordinates for Contacts 1 and 2; the second and third of Contacts 0 to 3) clinically used for symptomatic control in the OCD patients (Fig. 5).

#### Anterior–posterior STN and decisional impulsivity

**Figure 7:**
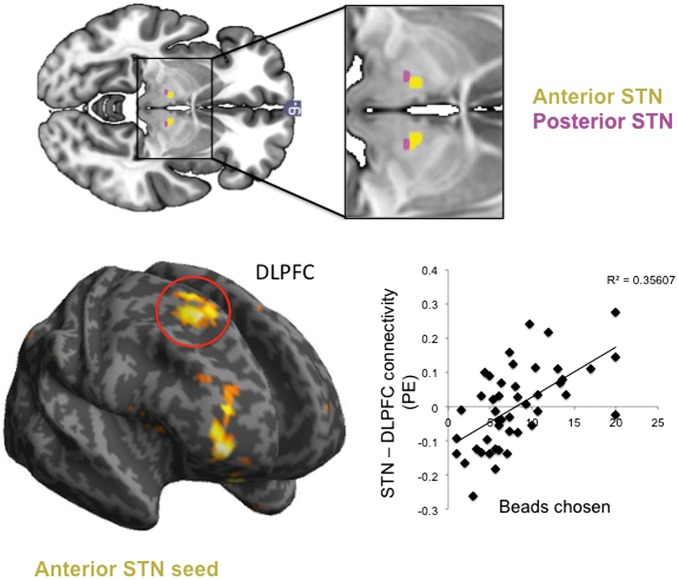
**Anterior and posterior STN and decisional impulsivity.***Top*: The anterior (yellow) and posterior (pink) STN seeds are shown. *Bottom*: Anterior STN seed-to-whole brain functional connectivity maps were correlated with the primary outcome measure from the Beads Task, the number of beads drawn prior to a decision. The scatter plot shows the parameter estimates (PE) for the correlation in the STN with beads chosen in the right dorsolateral prefrontal cortex (DLPFC). Displayed at *P* < 0 .005 whole brain uncorrected for illustration on standard MNI template.

The behavioural relevance of subregions of STN were examined. Of the 154 healthy volunteers who underwent functional MRI, 45 completed the Beads Task outside the scanner (27 female, age = 24.267 ± 5.663, Beads = 8.063 ± 4.824) and 76 completed the Monetary Choice Questionnaire (43 female, age = 28.474 ± 11.910, K = 0.013 ± 0.019, log10 K = 1.244 ± 0.589). Number of beads positively correlated with anterior STN connectivity with right dlPFC (*P* = 0.015, K = 128, Z = 4.33, *x y z* = 20 21 52) and right anterolateral PFC (*P* < 0.001, K = 47, Z = 4.62, *x y z* = 38 56 − 2), confirmed with small volume corrected FWE (*P* < 0.05 for dlPFC: *P* = 0.012, Z = 4.33) (Fig. 7). Number of beads negatively correlated with posterior STN connectivity with temporal cortex (*P* = 0.016, K = 124, Z = 3.90, *x y z* = 57 − 55 10). Log10 K-value positively correlated with posterior STN and SMA connectivity (*P* = 0.003, Z = 4.34, *x y z* = 3 3 75) with no correlations with anterior STN connectivity.

## Discussion

We show that stimulation of the anterior associative-limbic STN in OCD subjects increases decisional impulsivity. STN DBS decreases evidence accumulation (hence increasing impulsivity) in a probabilistic inference task relative to off DBS with greater evidence accumulation in OCD subjects off DBS relative to healthy controls. Notably STN DBS improves reflection impulsivity in OCD subjects closer towards healthy controls, thus emphasizing its adaptive nature. The checking behaviours usually associated with OCD, although intended to reduce uncertainty, instead increases uncertainty and doubt and impairs memory confidence (van den Hout and Kindt, 2003*a*, *b*; Coles *et al.*, 2006; Radomsky *et al.*, 2006; Boschen and Vuksanovic, 2007; Hermans *et al.*, 2008). However, under STN stimulation, OCD subjects might be less likely to check for evidence (e.g. be less likely to act on the obsessive fear that the stove was left on with repeated checking) with no difference in confidence (e.g. doubt or subjective uncertainty) or probability (e.g. objective uncertainty) at the time of decision. STN DBS targeting the anterior STN also increases impulsive choice (enhancing delay discounting) relative to off DBS. Although this measure did not differ from healthy controls, the direction of effect was to increase delay discounting further away from healthy controls, further emphasizing a dissociation between the two tasks. In a separate study in healthy controls, lower evidence accumulation (greater impulsivity) was associated with lower resting state functional connectivity between the anterior STN and dlPFC in healthy controls emphasizing differential functioning of anterior associative-limbic and posterior motor STN.

We further replicate in humans tracing studies performed in the non-human primate of prefrontal hyperdirect connectivity with subregions of the STN (Haynes and Haber, 2013). In particular, we show using resting state functional connectivity in a relatively large sample of healthy humans that limbic ventral striatum, prefrontal associative [dlPFC (BA 9, 46) and dACC (BA 24)] showed greater connectivity with bilateral anterior STN whereas SMA (caudal BA 6) and M1 had greater connectivity with bilateral posterior STN. In contrast, the nodes of the ‘stopping’ network were more difficult to localize with pre-SMA (rostro-medial BA 6) peaks across both anterior and posterior STN, consistent with rostral BA 6 projections in the non-human primate occurring more caudally than prefrontal projections but overlapping with dlPFC (BA46) projections (projections from the right IFC were not shown in the primate study). We further showed unilateral functional connectivity of bilateral ventral striatum to right anterior ventromedial STN. This finding is highly consistent with findings of emotive auditory stimulation evoked activity localized specifically to the right ventral non-oscillatory portion of the right STN and not to the left STN or dorsal regions (Eitan *et al.*, 2013). In general, peak connectivity of the right STN appeared to have greater anterior-posterior specificity corresponding to function with limbic and associative peaks in anterior and motor peaks in posterior STN. Such specificity was less clear for the left STN (e.g. for putamen and pre-SMA). We further highlight the anterior and ventral position of the actual effective contacts (Contacts 1 and 2) for clinical symptomatic improvement in the OCD patients. Together these findings highlight dissociable functioning of anterior limbic-associative and posterior motor STN and support lateralization of limbic functioning of the right STN (Eitan *et al.*, 2013).

### Neural mechanisms

This study focuses on evidence accumulation during probabilistic inference. STN DBS is commonly associated with hastened responding with a greater number of errors during response conflict in which a prepotent bias must be inhibited and an alternate competing response selected (Jahanshahi *et al.*, 2000; Schroeder *et al.*, 2002; Frank *et al.*, 2007; Green *et al.*, 2013). Greater conflict increases the decision threshold for evidence accumulation associated with increased medial prefrontal theta activity correlating with conflict-induced slowing (Cavanagh *et al.*, 2011) coupled with an increase in cortico-STN coherence (Zavala *et al.*, 2014). Similarly, STN single unit activity also increases as a function of conflict (Zaghloul *et al.*, 2012). In contrast, STN DBS decreases medial prefrontal theta activity and reverses the relationship between theta activity and conflict-induced slowing (Cavanagh *et al.*, 2011) and also impairs conflict-related performance on the Stroop interference task in the medial prefrontal cortex (dorsal cingulate) and ventral striatum (Schroeder *et al.*, 2002). Thus evidence accumulation in the context of conflict implicates mesial prefrontal and STN connectivity. In tasks such as the Random Dot Motion Task or probabilistic choice task with continuous outcomes (e.g. reaction time), evidence accumulation can be modelled using drift diffusion models. Decision thresholds, and hence the amount of evidence accumulated modelled in this manner have been shown to decrease with DBS targeting the motor STN in Parkinson’s disease patients (Frank *et al.*, 2007). In contrast, evidence accumulation obtained in a discrete explicit manner such as the Beads Task cannot be modelled using drift diffusion modelling. Our findings extend these reports of decreased evidence accumulation in the context of conflict to show that STN DBS of the limbic-associative STN decreases evidence accumulation during probabilistic inference.

Our results suggest specificity of the anterior limbic-associative STN in evidence accumulation during probabilistic inference and in delay discounting. In patients with Parkinson’s disease who have undergone STN DBS targeting the motor STN, no differences were observed in evidence accumulation with the Beads Task from Parkinson’s disease controls (Djamshidian *et al.*, 2013*b*). STN DBS improves delay discounting in rodents (Winstanley *et al.*, 2005; Uslaner and Robinson, 2006) with no clear effect in patients with Parkinson’s disease who have undergone DBS targeting the motor STN (Evens *et al.*, 2015). Our findings in OCD subjects with DBS targeting the limbic-associative STN and in functional connectivity in healthy controls of limbic-associative but not motor STN seeds highlights a dissociation in function between the limbic-associative and motor STN on decisional impulsivity.

Reflection impulsivity as tested in this probabilistic inference task includes several elements such as evidence accumulation, integration and decision in the context of probabilistic uncertainty (Stern *et al.*, 2010; Furl and Averbeck, 2011). In healthy controls, we show that the limbic-associative and not the motor STN had increased resting state functional connectivity with right dlPFC as a function of greater evidence accumulation. We have previously shown that less evidence accumulation (higher impulsivity) in the Beads Task is associated with lower volumes in the dlPFC, lateral parietal cortex and insular cortices (Banca *et al.*, 2015). The dlPFC is relevant during both evidence-seeking and the decision phase in a functional MRI study of the Beads Task (Furl and Averbeck, 2011). In another functional MRI study focusing on evidence accumulation using differing proportions of coloured cards, greater uncertainty during decision execution was associated with greater activity in lateral frontal and parietal activity (Stern *et al.*, 2010). Similarly, in a study involving a series of rapidly shifting shapes in which subjects must decide the likely shape, decision in the context of uncertainty was associated with greater dlPFC and posterior parietal activity (Huettel *et al.*, 2005). Although the dlPFC has also been implicated in working memory our version of the Beads Task controlled for working memory. Thus, our findings suggest that active evidence accumulation under probabilistic uncertainty implicates the right dlPFC and anterior STN whereas evidence accumulation under conflict implicates mesial PFC and STN.

That delayed discounting was increased with STN DBS contrasts with rodent STN lesion studies showing a decrease in delay discounting (Winstanley *et al.*, 2005; Uslaner and Robinson, 2006) and in STN DBS in patients with Parkinson’s disease targeting the motor STN showing no differences in delay discounting (Evens *et al.*, 2015; Seinstra *et al.*, 2016). That we observe the opposite effect may be related to specific targeting of limbic-associative STN or a patient group-specific effect in OCD subjects. This increase may also be associated with several underlying mechanisms including either greater sensitivity to the incentive of the immediate reward, greater temporal devaluation of the delayed reward, or greater intolerance of either the delay or the uncertainty associated with the delayed reward. There was no reward incentive or feedback associated with the Beads Task and no differences in probability (objective uncertainty) or confidence (subjective uncertainty) as a function of DBS.

Although there was no behavioural correlation between reflection impulsivity and delay discounting, there are several plausible explanations that might link both forms of impulsivity. STN DBS targeting the anterior STN might increase non-specific delay intolerance hence resulting in impulsive choices. STN DBS has also been shown to encode the cost-benefit ratio in the context of action value and effort (Zenon *et al.*, 2016). Both the accumulation of more evidence, which requires greater cognitive effort and time (without a reward incentive), and the prospect of a delayed outcome in choice may similarly be calculated as a cost. Alternatively, the role of contextual uncertainty may be relevant with STN DBS influencing the capacity to integrate and map future actions onto rewards in the context of uncertainty (Averbeck *et al.*, 2013). This might explain the shift towards more impulsive choices relative to larger delayed but potentially more uncertain choices, and more rapid decisions in the face of probabilistic uncertainty and be consistent with premature responding in the context of uncertainty.

### Evidence accumulation and symptom severity

We show that OCD subjects off DBS have elevated evidence accumulation relative to healthy controls. STN DBS was also associated with a negative correlation between the severity of preoperative OCD symptoms and lower objective probability at the time of decision. This relationship was explained by subjects with more severe preoperative OCD symptoms making decisions on DBS with either low evidence accumulation (high impulsivity) or high evidence accumulation (low impulsivity), both of which are associated with lower objective probabilities. These findings have several implications. That the preoperative severity of OCD rather than the current OCD severity influences cognitive function on DBS suggests a role for underlying trait effects. Thus, those with a severe history of OCD may have different underlying cognitive strategies that might explain this dichotomy with either enhanced tendency to jump to conclusions with limited evidence or to be overly cautious in checking for evidence.

The literature in OCD subjects in the general population is mixed. The Beads Task was originally designed to assess decision-making in OCD with the number of beads or evidence accumulated has been shown to be higher in OCD (Fear and Healey, 1997; Pelissier and O'Connor, 2002), although these findings are not always consistent (Volans, 1976) or only significant after controlling for neuroticism (Jacobsen *et al.*, 2012). Our findings of dichotomous cognitive styles may explain inconsistencies in the literature. OCD subjects who undergo DBS may also represent a different subgroup from those in the general population either as a function of severity or the willingness to undergo a major neurosurgical procedure.

### Implications for Parkinson’s disease

These findings have clinical implications for STN DBS in Parkinson’s disease (Castrioto *et al.*, 2014). STN DBS and the capacity to decrease dopaminergic medications can be effective in the management of dopaminergic medication-related impulse control disorders (ICDs). Although not all retrospective studies demonstrate an improvement (Moum *et al.*, 2012), improvement of impulse control disorders has been shown in the long term in prospective studies (Lhommee *et al.*, 2012; Amami *et al.*, 2015). However, in the postoperative period, rare new onset ICDs (e.g. gambling disorder) can occur or specific behaviours may be more likely to occur or not improve (e.g. binge eating) (Zahodne *et al.*, 2011). The right ventral STN has been shown to be associated with encoding of emotional stimuli in Parkinson’s disease (Eitan *et al.*, 2013). Furthermore, patients with Parkinson’s disease with preoperative ICDs show enhanced low frequency oscillatory activity in ventral limbic STN whereas those with preoperative levodopa-induced dyskinesias show enhanced activity in dorsal motor STN with differential coherence with associative and motor prefrontal regions, respectively (Rodriguez-Oroz *et al.*, 2011). One possible mechanism underlying these postoperative behaviours may be related to an interaction between underlying individual vulnerability and STN DBS localization influencing limbic and associative rather than motor territories.

### Limitations

Although the sample size was small, we note that the largest published randomized controlled trial study of STN DBS in OCD involved 16 patients (Mallet *et al.*, 2008). Furthermore, using a within-subject design to assess DBS effects reduces variability and allows for studies with smaller sample sizes. Although we only tested healthy volunteers once and OCD patients twice, this might be a minor issue, as there was no order effect.

## Conclusion

We highlight the role of the anterior limbic-associative STN in decisional impulsivity. Differential engagement of limbic-associative versus motor STN in decisional impulsivity is of direct relevance to target localization in psychiatric and neurological disorders.

## Abbreviations

dACC: dorsal cingulate
DBS: deep brain stimulation
dlPFC: dorsolateral prefrontal cortex
OCD: obsessive compulsive disorder
SMA: supplementary motor area
STN: subthalamic nucleus
YBOCS: Yale Brown Obsessive Compulsive Scale

## References

[aww309-B1] Amami P, Dekker I, Piacentini S, Ferre F, Romito LM, Franzini A, Impulse control behaviours in patients with Parkinson’s disease after subthalamic deep brain stimulation: de novo cases and 3-year follow-up. J Neurol Neurosurg Psychiatry 2015; 86: 562–4. 25012201 10.1136/jnnp-2013-307214

[aww309-B2] Averbeck BB, Djamshidian A, O’Sullivan SS, Housden CR, Roiser JP, Lees AJ Uncertainty about mapping future actions into rewards may underlie performance on multiple measures of impulsivity in behavioral addiction: evidence from Parkinson’s disease. Behav Neurosci 2013; 127: 245–55. 23565936 10.1037/a0032079 PMC3935250

[aww309-B69] Baek K, Morris LS, Kundu P Disrupted resting-state brain network properties in obesity: decreased global and putaminal cortico-striatal network efficiency. Psychol Med 2016: 1–12. 10.1017/S0033291716002646 PMC5426347 27804899

[aww309-B3] Banca P, Lange I, Worbe Y, Howell NA, Irvine M, Harrison NA, Reflection impulsivity in binge drinking: behavioural and volumetric correlates. Addict Biol 2016; 21: 504–15. 25678093 10.1111/adb.12227 PMC4766871

[aww309-B4] Banca P, Vestergaard MD, Rankov V, Baek K, Mitchell S, Lapa T, Evidence accumulation in obsessive-compulsive disorder: the role of uncertainty and monetary reward on perceptual decision-making thresholds. Neuropsychopharmacology 2015; 40: 1192–202. 25425323 10.1038/npp.2014.303 PMC4349497

[aww309-B5] Boschen MJ, Vuksanovic D Deteriorating memory confidence, responsibility perceptions and repeated checking: comparisons in OCD and control samples. Behav Res Ther 2007; 45: 2098–109. 17459333 10.1016/j.brat.2007.03.009

[aww309-B6] Castrioto A, Lhommee E, Moro E, Krack P Mood and behavioural effects of subthalamic stimulation in Parkinson’s disease. Lancet Neurol 2014; 13: 287–305. 24556007 10.1016/S1474-4422(13)70294-1

[aww309-B7] Cavanagh JF, Wiecki TV, Cohen MX, Figueroa CM, Samanta J, Sherman SJ, Subthalamic nucleus stimulation reverses mediofrontal influence over decision threshold. Nat Neurosci 2011; 14: 1462–7. 21946325 10.1038/nn.2925 PMC3394226

[aww309-B8] Chabardes S, Polosan M, Krack P, Bastin J, Krainik A, David O, Deep brain stimulation for obsessive-compulsive disorder: subthalamic nucleus target. World Neurosurg 2013; 80: S31e31 8. 10.1016/j.wneu.2012.03.010 22469523

[aww309-B9] Coles ME, Radomsky AS, Horng B Exploring the boundaries of memory distrust from repeated checking: increasing external validity and examining thresholds. Behav Res Ther 2006; 44: 995–1006. 16174515 10.1016/j.brat.2005.08.001

[aww309-B10] Dar R Elucidating the mechanism of uncertainty and doubt in obsessive-compulsive checkers. J Behav Ther Exp Psychiatry 2004; 35: 153–63. 15210376 10.1016/j.jbtep.2004.04.006

[aww309-B11] Djamshidian A, O’Sullivan SS, Foltynie T, Aviles-Olmos I, Limousin P, Noyce A, Dopamine agonists rather than deep brain stimulation cause reflection impulsivity in Parkinson’s disease. J Parkinson’s Dis2013a; 3: 139–44. 23938343 10.3233/JPD-130178 PMC4205962

[aww309-B12] Djamshidian A, Sanotsky Y, Matviyenko Y, O’Sullivan SS, Sharman S, Selikhova M, Increased reflection impulsivity in patients with ephedrone-induced Parkinsonism. Addiction2013b; 108: 771–9. 23228208 10.1111/add.12080 PMC3938292

[aww309-B13] Eitan R, Shamir RR, Linetsky E, Rosenbluh O, Moshel S, Ben-Hur T, Asymmetric right/left encoding of emotions in the human subthalamic nucleus. Front Syst Neurosci 2013; 7: 69. 24194703 10.3389/fnsys.2013.00069 PMC3810611

[aww309-B14] Endrass T, Klawohn J, Schuster F, Kathmann N Overactive performance monitoring in obsessive-compulsive disorder: ERP evidence from correct and erroneous reactions. Neuropsychologia 2008; 46: 1877–87. 18514679 10.1016/j.neuropsychologia.2007.12.001

[aww309-B15] Evens R, Stankevich Y, Dshemuchadse M, Storch A, Wolz M, Reichmann H, The impact of Parkinson’s disease and subthalamic deep brain stimulation on reward processing. Neuropsychologia 2015; 75: 11–19. 25976111 10.1016/j.neuropsychologia.2015.05.005

[aww309-B16] Fear CF, Healy D Probabilistic reasoning in obsessive-compulsive and delusional disorders. Psychol Med 1997; 27: 199–208. 9122300 10.1017/s0033291796004175

[aww309-B17] Fitzgerald KD, Welsh RC, Gehring WJ, Abelson JL, Himle JA, Liberzon I, Error-related hyperactivity of the anterior cingulate cortex in obsessive-compulsive disorder. Biol Psychiatry 2005; 57: 287–94. 15691530 10.1016/j.biopsych.2004.10.038

[aww309-B18] Frank MJ, Samanta J, Moustafa AA, Sherman SJ Hold your horses: impulsivity, deep brain stimulation, and medication in parkinsonism. Science 2007; 318: 1309–12. 17962524 10.1126/science.1146157

[aww309-B19] Furl N, Averbeck BB Parietal cortex and insula relate to evidence seeking relevant to reward-related decisions. J Neurosci 2011; 31: 17572–82. 22131418 10.1523/JNEUROSCI.4236-11.2011 PMC3474936

[aww309-B20] Gehring WJ, Himle J, Nisenson LG Action-monitoring dysfunction in obsessive-compulsive disorder. Psychol Sci 2000; 11: 1–6. 11228836 10.1111/1467-9280.00206

[aww309-B21] Grassi G, Pallanti S, Righi L, Figee M, Mantione M, Denys D, Think twice: impulsivity and decision making in obsessive-compulsive disorder. J Behav Addict 2015; 4: 263–72. 26690621 10.1556/2006.4.2015.039 PMC4712760

[aww309-B22] Green N, Bogacz R, Huebl J, Beyer AK, Kuhn AA, Heekeren HR Reduction of influence of task difficulty on perceptual decision making by STN deep brain stimulation. Curr Biol 2013; 23: 1681–4. 23932401 10.1016/j.cub.2013.07.001

[aww309-B23] Haynes WI, Haber SN The organization of prefrontal-subthalamic inputs in primates provides an anatomical substrate for both functional specificity and integration: implications for Basal Ganglia models and deep brain stimulation. J Neurosci 2013; 33: 4804–14. 23486951 10.1523/JNEUROSCI.4674-12.2013 PMC3755746

[aww309-B24] Hermans D, Engelen U, Grouwels L, Joos E, Lemmens J, Pieters G Cognitive confidence in obsessive-compulsive disorder: distrusting perception, attention and memory. Behav Res Ther 2008; 46: 98–113. 18076865 10.1016/j.brat.2007.11.001

[aww309-B25] Hershey T, Revilla FJ, Wernle A, Gibson PS, Dowling JL, Perlmutter JS Stimulation of STN impairs aspects of cognitive control in PD. Neurology 2004; 62: 1110–14. 15079009 10.1212/01.wnl.0000118202.19098.10

[aww309-B26] Hikosaka O, Isoda M Switching from automatic to controlled behavior: cortico-basal ganglia mechanisms. Trends Cogn Sci 2010; 14: 154–61. 20181509 10.1016/j.tics.2010.01.006 PMC2847883

[aww309-B27] Huettel SA, Song AW, McCarthy G Decisions under uncertainty: probabilistic context influences activation of prefrontal and parietal cortices. J Neurosci 2005; 25: 3304–11. 15800185 10.1523/JNEUROSCI.5070-04.2005 PMC6724903

[aww309-B28] Jaafari N, Aouizerate B, Tignol J, El-Hage W, Wassouf I, Guehl D, The relationship between insight and uncertainty in obsessive-compulsive disorder. Psychopathology 2011; 44: 272–6. 21546788 10.1159/000323607

[aww309-B100] Jacobsen P, Freeman D, Salkovskis P Reasoning bias and belief conviction in obsessive-compulsive disorder and delusions: jumping to conclusions across disorders. Br J Clin Psychol 2012; 51: 84–99. 22268543 10.1111/j.2044-8260.2011.02014.x

[aww309-B29] Jahanshahi M, Ardouin CM, Brown RG, Rothwell JC, Obeso J, Albanese A, The impact of deep brain stimulation on executive function in Parkinson’s disease. Brain 2000; 123(Pt 6): 1142–54. 10825353 10.1093/brain/123.6.1142

[aww309-B30] Johannes S, Wieringa BM, Nager W, Rada D, Dengler R, Emrich HM, Discrepant target detection and action monitoring in obsessive-compulsive disorder. Psychiatry Res 2001; 108: 101–10. 11738544 10.1016/s0925-4927(01)00117-2

[aww309-B31] Kashyap H, Kumar JK, Kandavel T, Reddy YC Neuropsychological functioning in obsessive-compulsive disorder: are executive functions the key deficit?. Compr Psychiatry 2013; 54: 533–40. 23419731 10.1016/j.comppsych.2012.12.003

[aww309-B32] Kiehl KA, Liddle PF, Hopfinger JB Error processing and the rostral anterior cingulate: an event-related fMRI study. Psychophysiology 2000; 37: 216–23. 10731771

[aww309-B33] Kundu P, Brenowitz ND, Voon V, Worbe Y, Vertes PE, Inati SJ, Integrated strategy for improving functional connectivity mapping using multiecho fMRI. Proc Natl Acad Sci USA 2013; 110: 16187–92. 24038744 10.1073/pnas.1301725110 PMC3791700

[aww309-B34] Lhommee E, Klinger H, Thobois S, Schmitt E, Ardouin C, Bichon A, Subthalamic stimulation in Parkinson’s disease: restoring the balance of motivated behaviours. Brain 2012; 135: 1463–77. 22508959 10.1093/brain/aws078

[aww309-B35] Mallet L, Polosan M, Jaafari N, Baup N, Welter ML, Fontaine D, Subthalamic nucleus stimulation in severe obsessive-compulsive disorder. N Engl J Med 2008; 359: 2121–34. 19005196 10.1056/NEJMoa0708514

[aww309-B36] Marsh R, Horga G, Parashar N, Wang Z, Peterson BS, Simpson HB Altered activation in fronto-striatal circuits during sequential processing of conflict in unmedicated adults with obsessive-compulsive disorder. Biol Psychiatry 2014; 75: 615–22. 23489416 10.1016/j.biopsych.2013.02.004 PMC3722261

[aww309-B37] Menzies L, Achard S, Chamberlain SR, Fineberg N, Chen CH, del Campo N, Neurocognitive endophenotypes of obsessive-compulsive disorder. Brain 2007; 130: 3223–36. 17855376 10.1093/brain/awm205

[aww309-B38] Morris LS, Kundu P, Baek K, Irvine MA, Mechelmans DJ, Wood J, Jumping the gun: mapping neural correlates of waiting impulsivity and relevance across alcohol misuse. Biol Psychiatry 2016; 79: 499–507. 26185010 10.1016/j.biopsych.2015.06.009 PMC4764648

[aww309-B39] Morris LS, Kundu P, Dowell N, Mechelmans D, Favre P, Irvine MA, Fronto-striatal organization: defining functional and microstructural substrates of behavioural flexibility. Cortex 2016; 74: 118–33. 26673945 10.1016/j.cortex.2015.11.004 PMC4729321

[aww309-B40] Moum SJ, Price CC, Limotai N, Oyama G, Ward H, Jacobson C, Effects of STN and GPi deep brain stimulation on impulse control disorders and dopamine dysregulation syndrome. PLoS One 2012; 7: e29768. 22295068 10.1371/journal.pone.0029768 PMC3266249

[aww309-B41] Najmi S, Hindash AC, Amir N Executive control of attention in individuals with contamination-related obsessive-compulsive symptoms. Depress Anxiety 2010; 27: 807–12. 20821800 10.1002/da.20703 PMC3998819

[aww309-B42] Nambu A, Tokuno H, Takada M Functional significance of the cortico-subthalamo-pallidal ‘hyperdirect’ pathway. Neurosci Res 2002; 43: 111–17. 12067746 10.1016/s0168-0102(02)00027-5

[aww309-B43] Page LA, Rubia K, Deeley Q, Daly E, Toal F, Mataix-Cols D, A functional magnetic resonance imaging study of inhibitory control in obsessive-compulsive disorder. Psychiatry Res 2009; 174: 202–9. 19906516 10.1016/j.pscychresns.2009.05.002

[aww309-B44] Pelissier MC, O’Connor KP Deductive and inductive reasoning in obsessive-compulsive disorder. Br J Clin Psychol 2002; 41: 15–27. 11931675 10.1348/014466502163769

[aww309-B45] Pinto A, Steinglass JE, Greene AL, Weber EU, Simpson HB Capacity to delay reward differentiates obsessive-compulsive disorder and obsessive-compulsive personality disorder. Biol Psychiatry 2014; 75: 653–59. 24199665 10.1016/j.biopsych.2013.09.007 PMC3969772

[aww309-B46] Radomsky AS, Gilchrist PT, Dussault D Repeated checking really does cause memory distrust. Behav Res Ther 2006; 44: 305–16. 15890313 10.1016/j.brat.2005.02.005

[aww309-B47] Ray NJ, Jenkinson N, Brittain J, Holland P, Joint C, Nandi D, The role of the subthalamic nucleus in response inhibition: evidence from deep brain stimulation for Parkinson’s disease. Neuropsychologia 2009; 47: 2828–34. 19540864 10.1016/j.neuropsychologia.2009.06.011

[aww309-B48] Rodriguez-Oroz MC, Lopez-Azcarate J, Garcia-Garcia D, Alegre M, Toledo J, Valencia M, Involvement of the subthalamic nucleus in impulse control disorders associated with Parkinson’s disease. Brain 2011; 134: 36–49. 21059746 10.1093/brain/awq301

[aww309-B49] Rotge JY, Clair AH, Jaafari N, Hantouche EG, Pelissolo A, Goillandeau M, A challenging task for assessment of checking behaviors in obsessive-compulsive disorder. Acta Psychiatr Scand 2008; 117: 465–73. 18331575 10.1111/j.1600-0447.2008.01173.x

[aww309-B50] Sarig S, Dar R, Liberman N Obsessive-compulsive tendencies are related to indecisiveness and reliance on feedback in a neutral color judgment task. J Behav Ther Exp Psychiatry 2012; 43: 692–7. 21983353 10.1016/j.jbtep.2011.09.012

[aww309-B51] Schroeder U, Kuehler A, Haslinger B, Erhard P, Fogel W, Tronnier VM, Subthalamic nucleus stimulation affects striato-anterior cingulate cortex circuit in a response conflict task: a PET study. Brain 2002; 125: 1995–2004. 12183345 10.1093/brain/awf199

[aww309-B52] Seinstra M, Wojtecki L, Storzer L, Schnitzler A, Kalenscher T No effect of subthalamic deep brain stimulation on intertemporal decision-making in Parkinson patients. eNeuro 2016; 23: 3. 10.1523/ENEURO.0019-16.2016 PMC4876489 27257622

[aww309-B53] Sheehan DV, Lecrubier Y, Sheehan KH, Amorim P, Janavs J, Weiller E, The Mini-International Neuropsychiatric Interview (M.I.N.I.): the development and validation of a structured diagnostic psychiatric interview for DSM-IV and ICD-10. J Clin Psychiatry 1998; 59(Suppl 20): 22–33. 9881538

[aww309-B54] Sohn SY, Kang JI, Namkoong K, Kim SJ Multidimensional measures of impulsivity in obsessive-compulsive disorder: cannot wait and stop. PLoS One 2014; 9: e111739. 25372136 10.1371/journal.pone.0111739 PMC4221112

[aww309-B55] Stern ER, Gonzalez R, Welsh RC, Taylor SF Updating beliefs for a decision: neural correlates of uncertainty and underconfidence. J Neurosci 2010; 30: 8032–41. 20534851 10.1523/JNEUROSCI.4729-09.2010 PMC2896864

[aww309-B56] Stern ER, Welsh RC, Gonzalez R, Fitzgerald KD, Abelson JL, Taylor SF Subjective uncertainty and limbic hyperactivation in obsessive-compulsive disorder. Hum Brain Mapp 2013; 34: 1956–70. 22461182 10.1002/hbm.22038 PMC5289818

[aww309-B57] Ursu S, Stenger VA, Shear MK, Jones MR, Carter CS Overactive action monitoring in obsessive-compulsive disorder: evidence from functional magnetic resonance imaging. Psychol Sci 2003; 14: 347–53. 12807408 10.1111/1467-9280.24411

[aww309-B58] Uslaner JM, Robinson TE Subthalamic nucleus lesions increase impulsive action and decrease impulsive choice—mediation by enhanced incentive motivation?. Eur J Neurosci 2006; 24: 2345–54. 17074055 10.1111/j.1460-9568.2006.05117.x

[aww309-B59] van den Hout M, Kindt M Phenomenological validity of an OCD-memory model and the remember/know distinction. Behav Res Ther2003a; 41: 369–78. 12600406 10.1016/s0005-7967(02)00097-9

[aww309-B60] van den Hout M, Kindt M Repeated checking causes memory distrust. Behav Res Ther2003b; 41: 301–16. 12600401 10.1016/s0005-7967(02)00012-8

[aww309-B61] Volans PJ Styles of decision-making and probability appraisal in selected obsessional and phobic patients. Br J Soc Clin Psychol 1976; 15: 305–17. 1009292 10.1111/j.2044-8260.1976.tb00038.x

[aww309-B62] Voon V, Dalley JW Translatable and back-translatable measurement of impulsivity and compulsvity: convergent and divergent processes. In: Robbins TW, Sahakian BJ (eds). Current topics in behavioural neuroscience; Springer, New York, 2015. 10.1007/7854_2015_5013 27418067

[aww309-B63] Whitfield-Gabrieli S, Nieto-Castanon A Conn: a functional connectivity toolbox for correlated and anticorrelated brain networks. Brain Connect 2012; 2: 125–41. 22642651 10.1089/brain.2012.0073

[aww309-B64] Winstanley CA, Baunez C, Theobald DE, Robbins TW Lesions to the subthalamic nucleus decrease impulsive choice but impair autoshaping in rats: the importance of the basal ganglia in Pavlovian conditioning and impulse control. Eur J Neurosci 2005; 21: 3107–16. 15978020 10.1111/j.1460-9568.2005.04143.x

[aww309-B65] Zaghloul KA, Weidemann CT, Lega BC, Jaggi JL, Baltuch GH, Kahana MJ Neuronal activity in the human subthalamic nucleus encodes decision conflict during action selection. J Neurosci 2012; 32: 2453–60. 22396419 10.1523/JNEUROSCI.5815-11.2012 PMC3296967

[aww309-B66] Zahodne LB, Susatia F, Bowers D, Ong TL, Jacobson CE, Okun MS, Binge eating in Parkinson’s disease: prevalence, correlates and the contribution of deep brain stimulation. J Neuropsychiatry Clin Neurosci 2011; 23: 56–62. 21304139 10.1176/appi.neuropsych.23.1.56 PMC3075093

[aww309-B67] Zavala BA, Tan H, Little S, Ashkan K, Hariz M, Foltynie T, Midline frontal cortex low-frequency activity drives subthalamic nucleus oscillations during conflict. J Neurosci 2014; 34: 7322–33. 24849364 10.1523/JNEUROSCI.1169-14.2014 PMC4028502

[aww309-B68] Zenon A, Duclos Y, Carron R, Witjas T, Baunez C, Regis J, The human subthalamic nucleus encodes the subjective value of reward and the cost of effort during decision-making. Brain 2016; 139: 1830–43. 27190012 10.1093/brain/aww075 PMC4937992

